# Polymorphism of Sex Determination Amongst Wild Populations Suggests its Rapid Turnover Within the Nile Tilapia Species

**DOI:** 10.3389/fgene.2022.820772

**Published:** 2022-05-17

**Authors:** Cécile Triay, Maxime Courcelle, Pierre Caminade, Etienne Bezault, Jean-François Baroiller, Thomas D. Kocher, Helena D’Cotta

**Affiliations:** ^1^ UMR116-Institut des Sciences de l’Evolution de Montpellier, Centre de Coopération Internationale en Recherche Agronomique pour le Développement, Campus International Baillarguet, Montpellier, France; ^2^ UMR-Institut des Sciences de l’Evolution de Montpellier, Centre National de la Recherche Scientifique, Institut de Recherche Pour le Développement, Ecole Pratique des Hautes Etudes, University of Montpellier, Montpellier, France; ^3^ UMR BOREA, CNRS-7208/MNHN/UPMC/IRD-207/UCN/UA, Université des Antilles, Guadeloupe, France; ^4^ Department of Biology, University of Maryland, College Park, MD, United States

**Keywords:** sex chromosome, sex determination, Y-haplotype, AMH, duplication, populations

## Abstract

Sex-determining regions have been identified in the Nile tilapia on linkage groups (LG) 1, 20 and 23, depending on the domesticated strains used. Sex determining studies on wild populations of this species are scarce. Previous work on two wild populations, from Lake Volta (Ghana) and from Lake Koka (Ethiopia), found the sex-determining region on LG23. These populations have a Y-specific tandem duplication containing two copies of the Anti-Müllerian Hormone *amh* gene (named *amhY* and *amhΔY*). Here, we performed a whole-genome short-reads analysis using male and female pools on a third wild population from Lake Hora (Ethiopia). We found no association of sex with LG23, and no duplication of the *amh* gene. Furthermore, we found no evidence of sex linkage on LG1 or on any other LGs. Long read whole genome sequencing of a male from each population confirmed the absence of a duplicated region on LG23 in the Lake Hora male. In contrast, long reads established the structure of the Y haplotype in Koka and Kpandu males and the order of the genes in the duplicated region. Phylogenies constructed on the nuclear and mitochondrial genomes, showed a closer relationship between the two Ethiopian populations compared to the Ghanaian population, implying an absence of the LG23Y sex-determination region in Lake Hora males. Our study supports the hypothesis that the *amh* region is not the sex-determining region in Hora males. The absence of the Y *amh* duplication in the Lake Hora population reflects a rapid change in sex determination within Nile tilapia populations. The genetic basis of sex determination in the Lake Hora population remains unknown.

## 1 Introduction

Genetic sex determination (GSD) inherited through sex chromosomes has been highly conserved and relatively stable in mammals and birds for millions of years (Cortez et al., 2014). In the majority of mammals, sex chromosomes are heteromorphic and follow an XX/XY system. The degenerated Y chromosome carries in most mammals the *Sry* gene, the master gene that triggers the development of male gonads (Sinclair et al., 1990). Birds in contrast have a derived heteromorphic ZW/ZZ system and in most cases ZW embryos become female. Testes develop in the presence of a double dose of the *dmrt1* gene on the Z chromosome (Smith et al., 2009; Graves, 2014). The mechanisms of sex determination evolve more rapidly in other vertebrates (Bachtrog et al., 2014). In reptiles, amphibians and fish, sex chromosomes and sex-determination genes often differ among even closely related species (Ezaz et al., 2009; Myosho et al., 2012; Jeffries et al., 2018). Temperature-dependent sex determination (TSD) is also widespread amongst reptiles (Holleley et al., 2016). Most fish species have a GSD dependent on single genes with a large effect carried by sex chromosomes, although some depend on interactions between several genes (Capel, 2017). The environment, particularly temperature, can also determine fish sex (Environmental Sex Determination = ESD) or influence sexual differentiation (Temperature Induced Sex Differentiation = TISD). In the case of temperature-sensitive GSD species, sex reversals can lead to mismatches between phenotypes and sexual genotypes (Baroiller and D'Cotta, 2016).

Sex chromosomes evolve in response to unique patterns of mutation, selection and recombination (Bachtrog et al., 2011). In the classical model of sex chromosome evolution, a new sex chromosome originates when an autosome acquires a sex-determining mutation (e.g., a new Y). If this new sex locus is linked to male-beneficial alleles (sexually antagonistic alleles), it is expected to increase in frequency (Rice, 1987; Charlesworth et al., 2005; van Doorn and Kirkpatrick, 2010). Selection favors suppression of recombination to maintain linkage disequilibrium between the new Y locus and male-beneficial alleles at nearby loci. The non-recombining region may then accumulate a variety of mutations, inversions, and repetitive elements (Kirkpatrick 2010; Bachtrog, 2013; Blaser et al., 2014). Over time the X and Y may become morphologically distinct (Bachtrog, 2013; Abbott et al., 2017). In contrast, 95% of teleost fishes have cytogenetically homomorphic sex chromosomes (Arai, 2011), implying that recombination suppression is probably recent. This is most likely due to the high turnover rates of teleost sex chromosomes, so that the differentiation of the sex chromosomes is reinitiated (Sember et al., 2021). The differences between sexes may be limited to a single missense SNP, such as in pufferfish (Kamiya et al., 2012).

Genomic sequencing has shed light on the diversity of sex systems and sex chromosomes in fish lineages, emphasizing their plasticity with rapid turnover of teleost sex chromosomes and in many cases the existence of multiple sex chromosomes (Sember et al., 2021; Tao et al., 2021). There are frequent transitions in the sex chromosome system, even within the same genus (Cnaani et al., 2008; Takehana et al., 2008; Roberts et al., 2009; Böhne et al., 2019). Some sex-determining genes have been conserved but translocated into different chromosomes (Guiguen et al., 2018). For instance, in the ricefish, the sex-determination gene *dmY* (*also called dmrt1bY*) was found on linkage group (LG) 1 in the medaka *Oryzias latipes* (Matsuda et al., 2002; Nanda et al., 2002). However, in *O. luzonensis* the sex-determiner is a Y-linked copy of the *gsdf* gene located on LG12 (Myosho et al., 2012) and in other *Oryzias* it is *sox3* on LG10 (Takehana et al., 2014). A remarkable number of sex chromosome turnovers have occurred in the family Cichlidae, where over 20 sex-determiners have been identified on more than 17 different LGs during an adaptive radiation in East Africa (Gammerdinger & Kocher, 2018; El Taher et al., 2021). Sex determiners have evolved multiple times on the same chromosomes (e.g., on LG5, LG7 and LG19) (Böhne et al., 2019; El Taher et al., 2021). Differentiation of the sex chromosomes can be limited to a small region, or extend over the whole chromosome indicative of extensive recombination suppression (El Taher et al., 2021).

The high species diversity of teleost fish (approximately 27,000 living species) has been proposed to be related to the genomic plasticity of this clade (Helfman et al., 2009). Fishes have a higher rate of evolution and gene duplications than other vertebrates (Mank and Avise, 2009). Indeed, gene duplications are a substrate for the evolution of innovations by giving the duplicated copy (paralog) the possibility to partition the function of the original gene (subfunctionalization), or to acquire a new function (neofunctionalization) (Sandve et al., 2018). Several teleost sex-determining genes have emerged from gene duplication (Mank and Avise, 2009; Ortega-Recalde et al., 2020). *Dmy* of medaka is a duplicate of the male-differentiating *dmrt1* gene found on the autosomal LG9, that was translocated to LG1 (Matsuda et al., 2002; Nanda et al., 2002). In the Patagonian pejerrey (*Odontesthes hatcheri*) and the Northern pike (*Esox lucius*) duplicated copies of the *amh* gene have taken on the role of master sex-determiner (Hattori et al., 2012; Pan et al., 2019). Duplication of genes outside the sex pathway can also result in a major sex-determinant such as the *sdy* gene in salmonids, a neofunctionalized paralog of an immune-related gene (*irf9*) (Yano et al., 2012).

Nile tilapia have an XX/XY GSD that can be overridden at high temperatures to generate XX males (Baroiller et al., 2009). This environmental sensitivity, together with extensive hybridization among cultivated strains, has complicated the characterization of the sex chromosomes in tilapias. In some strains sex has been linked to LG1 (Cnaani et al., 2008; Gammerdinger et al., 2014; Palaiokostas et al., 2015) while others show sex linkage on LG23 (Eshel et al., 2014; Li et al., 2015; Wessels et al., 2017; Cáceres et al., 2019) or to both (Taslima et al., 2021). LG20 has also been linked to sex in the domesticated Manzala-Stirling strain (Palaiokostas et al., 2015). The causal gene(s) on LG1 has (ve) not yet been identified. In the Japanese domesticated strain of Nile tilapia, the *amh* gene on LG23 has been identified as the sex-determination gene, which evolved through a tandem duplication within the same LG. Hence, males have three *amh* copies: one on the X chromosome and two copies located in tandem on the Y chromosome, one with a missense SNP (*amhY*) and the other with an insertion that causes a premature stop codon (*amhΔY*) (Li et al., 2015). We previously found that sex in three wild populations, from Burkina Faso and Ghana (West Africa), and Ethiopia (East Africa) was linked to LG23 (Sissao et al., 2019; Triay et al., 2020). The sex-determining region of the Y chromosome contains an *amh* duplication estimated to be ∼51 kb using short reads from whole genome sequencing (WGS) and encompassing the adjacent *oaz1* and *dot1l* genes (Triay et al., 2020). However, we were not able to determine the order of the genes on the Y haplotype. We also identified differences in the *amhΔY* copy in the two wild populations compared with domesticated strains. *AmhΔY* in the Ethiopian Koka population lacks the 5 bp insertion which causes a premature stop codon (Triay et al., 2020). In addition, some *amh* markers that were sex-linked in the West African population did not discriminate between XX and XY individuals in the Ethiopian population (Sissao et al., 2019; Triay et al., 2020).

Here, we explore the genetic basis of sex determination in another Ethiopian population from Lake Hora, which is geographically and phylogenetically close to the population in Lake Koka. Both populations live in cold lakes (17–26°C) and belong to the subspecies *O. niloticus cancellatus*. We expected that the sex-linked duplication of the *amh* region on LG23 would also be present in Hora males. We used WGS to compare pools of Hora males and females, but failed to identify a sex-linked region. We then used long-read Nanopore sequencing to characterize the LG23 Y haplotype of males from the Kpandu (Ghana) and Koka (Ethiopia) populations and compared them to the corresponding LG23 region of a male from Lake Hora (Ethiopia). Lack of sex-associated SNP pattern on LG23 and Y specific structures and sequences of the duplicated *amh* region in Hora males, support the hypothesis that the *amh* region is not the sex-determining region in Hora males and suggests that turnover of the sex-determination system can occur extremely rapidly. Finally, we built phylogenetic trees to characterize the evolutionary history of these populations.

## 2 Materials and Methods

### 2.1 Fish Samples and DNA Extractions

Nile tilapia (*Oreochromis niloticus*) were caught in 2002 and 2003 from the Sudano-Sahelian basin in West Africa and from two populations in the Ethiopian Rift Valley basin of East Africa (Bezault, 2005). The Kpandu population belongs to the subspecies *O. niloticus niloticus* and was collected in Ghana from a dendritic expansion of the eastern side of Lake Volta where temperatures fluctuate between 27 and 32°C. The Ethiopian populations belong to the subspecies *O. niloticus cancellatus* and were collected from two cold-temperature (between 17 and 26°C) lakes in the Awash River basin in the Ethiopian highlands. Lake Koka is a large artificial reservoir of the Awash River of 255 km^2^ with an average depth of 9 m. Lake Hora is an isolated lake of about 1 km^2^ with a depth of up to 35 m. Fully mature fish were collected using seine nets and the phenotypic sex was then defined by external examination of the genital papilla (males have only one urogenital orifice whereas females have a genital opening and a urinary orifice; see Baroiller and D’Cotta, 2019). Fish were anaesthetized (but not sacrificed) with 2-phenoxyethanol to sample the fin clips which were preserved in 96–100% ethanol.

High-molecular weight genomic DNA (gDNA) was lysed from the fin clips in TNES-Urea buffer (4 M urea; 10 mM Tris-HCl, pH 7.5; 125 mM NaCl; 10 mM EDTA; 1% SDS) and extracted using the phenol-chloroform procedure. The gDNA was stored in Tris-EDTA buffer at -20°C. DNA was quantified first on a NanoDrop spectrophotometer (ThermoFisher, France) to estimate the absorbance ratios and the concentration was then measured on a Qubit 2.0 fluorometer (Invitrogen Carlsbad, United States). gDNA quality was also verified on a 0.8% agarose gel.

### 2.2 Whole Genome Short Reads Sequencing Analysis of Hora

#### 2.1.1 Libraries and Sequencing

Twenty WGS libraries were prepared at Macrogen (Seoul, Korea) from the gDNA of ten males and ten females gDNA that had been sonicated to obtain 550 bp fragments. All libraries were prepared using the TruSeq DNA PCR-Free kit (Illumina) except one male library where TruSeq Nano DNA Kit (Illumina) was used due to limited amounts of gDNA. Libraries were sequenced (150bp paired-end) in an S4 flow cell on a NovaSeq 6000 (Illumina, San Diego, CA).

#### 2.1.2 Data Processing

Raw data quality was checked using FastQC (0.11.7). Illumina TruSeq adapters were trimmed and short reads (<15bp) removed using *fastp* (version 0.21.0) (Chen et al., 2018). The fastq files were pooled according to the phenotypic sex of the samples, resulting in a pool of females reads (with 10 samples) and a pool of males reads (with 10 samples). The two pools were mapped to the latest *O. niloticus* reference genome available on NCBI (O_niloticus_UMD_NMBU, Genbank ID: GCA_001858045.3) using BWA mem mapper (version 0.7.15) (Li and Durbin, 2009). Sam files were then converted to Bam files and sorted using SAMtools (version 1.9) (Li et al., 2009) and read groups were added to the two pools using Picard AddOrReplaceReadGroups (2.7.0) (Broad Institute, 2019). The output files were then combined with SAMtools mpileup (version 1.9). Using Popoolation2 mpileup2sync.pl (version 1.2.2) (Kofler et al., 2011), the mpileup file was transformed to a synchronized file (sync). Finally, the sync file was processed with Sex_SNP_finder_GA.pl (Gammerdinger et al., 2014) to calculate the *F*st between males and females. This pipeline allowed us to identify sites that are fixed (or nearly fixed) in one sex and at intermediate frequencies in the other sex. The following parameters were used in Sex_SNP_finder: fixed_threshold = 0.9; minimum_polymorphic_frequency = 0.3; maximum_polymorphic_frequency = 0.7; minimum_read_depth = 10; maximum_read_depth = 100; minimum_read_count = 2; sex_SNP_finder_window_size = 10000. Finally, the *F*st were plotted on a manhattan plot using R and the qqman package (version 0.1.4) (Turner, 2018).

### 2.3 Library Construction for MinION Sequencing

One Kpandu male, one Koka male and one Hora male were sequenced using the MinION platform (Oxford Nanopore Technology). The male genomic DNA libraries were constructed from 3 to 4 µg of high molecular weight gDNA by MGX (Montpellier, France) using the Ligation Sequencing Kit 1D (ONT). Samples were fragmented, the ends repaired and adapters ligated. The flow cell FLO-MIN106 (flow cell 9.4.1) was charged twice, with a DNase treatment performed on the flow cell to reactivate the pores when half of the library was sequenced.

### 2.4 Subsetting of Read Datasets, Identification of Y Specific Reads and Assembly

**FIGURE 1:**
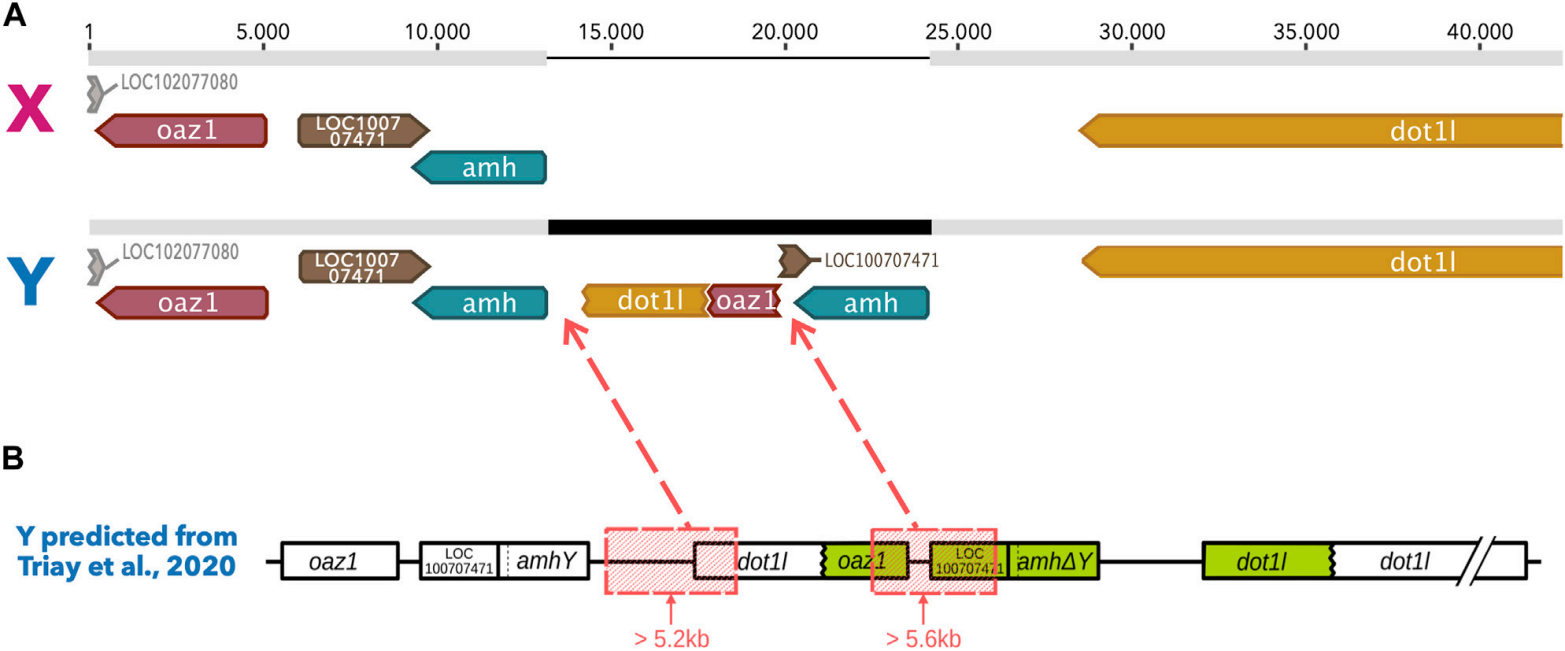
Alignment of both the X haplotype and the Y predicted haplotype built according to the descriptions of the tandem duplication in Triay et al., 2020 **. (A)** Geneious alignment of both the X haplotype from the reference genome and the Y theoretical haplotype. Grey bars represent homologous sequences between the X and the Y haplotypes whereas the black region in the Y haplotype represents Y specific regions that appeared due to a duplication of the *dot1l*, *oaz1,* LOC100707471 and *amh* genes. A chimera of the *dot1l* and *oaz1* genes is seen as the result of large deletions that occurred after the duplication. The second copy of the *amh* gene however is well conserved in this inserted region. **(B)** Y haplotype prediction published in Triay et al. (2020) with the position of the large deleted regions (in red boxes) removed from the tandem duplication to build the Y of the alignment.

Using the results of the Illumina short reads (2 × 150bp) whole genome sequencing published in Triay et al. (2020), we built an expected Y haplotype for the LG23 sex region (Figure 1). This expected Y haplotype is based on the sequence of the X haplotype of the reference genome (an XX female), to which the region corresponding to the duplication of the *oaz1* to *dot1l* genes was inserted according to the breakpoints described in Triay et al. (2020). We kept the nucleotides matching the small deletions (<500bp) found using the short read data in this expected haplotype, as we assumed the Nanopore reads to be long enough to cross short deletions. However, we deleted the sequences corresponding to the two large (>5 kb) deletions to obtain the final sequence of the theoretical Y.

Oxford Nanopore MinION raw reads were used to build a BLAST database (Camacho et al., 2009) against which were blasted the sequences of the *oaz1*, *amh* and *dot1l* genes extracted from the latest available *O. niloticus* XX female reference genome (O_niloticus_UMD_NMBU, Genbank ID: GCA_001858045.3). Since the first intron of the *dot1l* gene is very similar to the sequence of the unrelated *vasa* gene, we split the sequence of *dot1l* in order to remove this intron and limit its influences in the blast results. All raw reads for which at least one of the sequences presented blast hits were kept. This subset of reads was then trimmed for 23bp at the 3′ and 5′ ends to remove regions with low quality bases. This set of trimmed reads was then mapped onto the theoretical Y haplotype in order to identify Y specific reads according to the presence of Y specific indels described in Triay et al. (2020). We mainly focused on the deletion of about 275bp in length in *oaz1* shown to be Y specific in both Kpandu and Koka males (Triay et al., 2020). All reads presenting this deletion were thus labelled as “Y-specific” reads. Conversely, all other reads were labelled as “Non Y-specific.”

Reads of each subset (All Reads, Y specific, Non Y-specific) were *de novo* assembled using Canu (version 2.1.1) (Koren et al., 2017). The resulting contigs were automatically annotated using Geneious (version 9.0.5) (Kearse et al., 2012), based on the annotation of the reference genome for the defined region. Contigs that could not be annotated were checked using BLAST (version 2.11.0) and were removed when not belonging to the LG23 region of interest. This was the case mainly for the “Non Y-specific” subset of reads and when all reads were used to build *de novo* assemblies. This BLAST approach, used to identify genes belonging to the LG23 sex region, led us to keep some reads of the *oaz1b* gene (a paralog of *oaz1*) and *vasa* (of which part of the sequence is similar to the *dot1l* gene). However, contigs containing those genes were easily removed from the final set of contigs. Contigs that were confirmed to belong to LG23 were mapped to the *O. niloticus* XX female reference genome and to the Y theoretical haplotype using the Geneious assembler (version 9.0.5) with high sensitivity settings to check for the haplotype they corresponded to and whether the structure was the expected one.

In order to increase the contiguity of the Koka Y haplotype, we did a pairwise alignment of only the Y-specific reads using MAFFT (Katoh and Standley, 2013) with a scoring matrix of 100PAM/K = 2 and an automatically chosen algorithm. Among the Y-specific reads, some contained both the *amh* copies or both the *dot1l* copies. Thus, the pairwise alignment allowed us to compute a consensus sequence with the Y specific reads, increasing the length of the Y haplotype contig for the Koka male, even though the whole region could not be constructed using long reads assembly software. However, this method of consensus sequence with a low coverage of reads results in many ambiguities.

### 2.5 Alignments and Phylogeny

#### 2.5.1 Nuclear Phylogeny

The 42 loci used by Meyer et al. (2015) to construct their cichlid phylogeny were retrieved in the trimmed data of our wild populations using HMMER (version 3.3.2) (Wheeler and Eddy, 2013) (Supplementary Table S1). In order to maximize the accuracy of nucleotide sequences comparisons, we decided to use only the Illumina short reads data obtained from Hora in the current study, and those for Koka and Kpandu from our previous study (Triay et al., 2020). Because a B chromosome is suspected to be present in the WGS short reads from the female pool of Koka as well as in the male pool of Kpandu individuals, we selected for the phylogeny the Koka male pool, the Kpandu female pool and the Hora female pool. The targeted regions of the mapping alignments were converted from the BAM file to consensus fasta files after SNP calling using bcftools (version 1.9) (Narasimhan et al., 2016). For the two outgroups *Etia nguti* and *Hemichromis elongatus*, and for the *O. niloticus* reference genome, we used the fasta files available on NCBI (Genbank IDs: GCA_015106755.1, GCA_015108515.1 and GCA_001858045.3 respectively). When needed, we produced a reverse complement in order to obtain sequences in identical sense to the sequences of Meyer et al. (2015). The 42 sequences of all samples were then combined into a unique fasta and we added some of the species of Meyer et al. (2015). All sequences were then aligned using MAFFT (v7.475) (Katoh and Standley, 2013) and sites presenting more than 50% missing data among all species/samples, usually corresponding to species-specific insertions, were removed using trimAl (version 1.4. rev22) (Capella-Gutiérrez et al., 2009). Finally, a Maximum-Likelihood phylogeny was produced using IQtree (1.6.12) with 100 bootstraps for node support.

#### 2.5.2 Mitochondrial Phylogeny

We assembled the mitochondrial genomes from Koka, Hora and Kpandu *O. niloticus* wild populations obtained from the short reads of our Whole Genome Sequencing trimmed data using MitoFinder (version 1.4) (Allio et al., 2020). We used the reference mitochondrial genome as the basis for annotation (Genbank ID: GU238433.1). In addition, we used the closely related species *Haplochromis burtoni* as outgroup (NCBI ID: NC_027289.1). In order to use the same basis as the one used for gene annotation, we also ran the reference mitochondrial genome and *Haplochromis burtoni* through Mitofinder to extract individual genes. The sequences of the 13 mitochondrial protein-coding genes and the two ribosomal subunits (Supplementary Table S2) were then aligned using MAFFT (v7.475) (Katoh and Standley, 2013). A maximum-likelihood phylogeny was inferred from the concatenated sequence alignments using IQtree 1.6.12 (Nguyen et al., 2015) with 100 bootstraps for node support.

#### 2.5.3.Phylogeny of Sex Haplotypes

Because of the low coverage of long reads data and due to the high error rate of Oxford Nanopore MinION data, we could not use the Y haplotypes built from the *de novo* analyses. Thus, we focused on the *amh* gene region and more precisely on the LG23 region from 34,489,620 to 34,532,693 bp according to the XX reference genome and analyzed only female individuals. This corresponds to the region defined as carrying the major sex-determinant in previous studies (Li et al., 2015; Cáceres et al., 2019; Triay et al., 2020; Taslima et al., 2021). This region was extracted from Kpandu, Koka and Hora female mapping files using SAMtools (version 1.9) (Li et al., 2009). These regions were then converted to consensus sequences using Geneious (version 9.0.5) (Kearse et al., 2012). The sequences, along with fasta files of this region from *O. niloticus* and *O. aureus* were aligned using MAFFT (v7.475) (Katoh and Standley, 2013). The subsequent alignments were then used to build a phylogeny of Females X haplotypes with 100 bootstraps with IQtree (1.6.12) (Nguyen et al., 2015). The final visualization of all the trees (nuclear, mitochondrion and X haplotypes) were formatted using Geneious (version 9.0.5) (Kearse et al., 2012).

## 3 Results

### 3.1 Absence of Sex-Association on LG23 in Lake Hora

**FIGURE 2:**
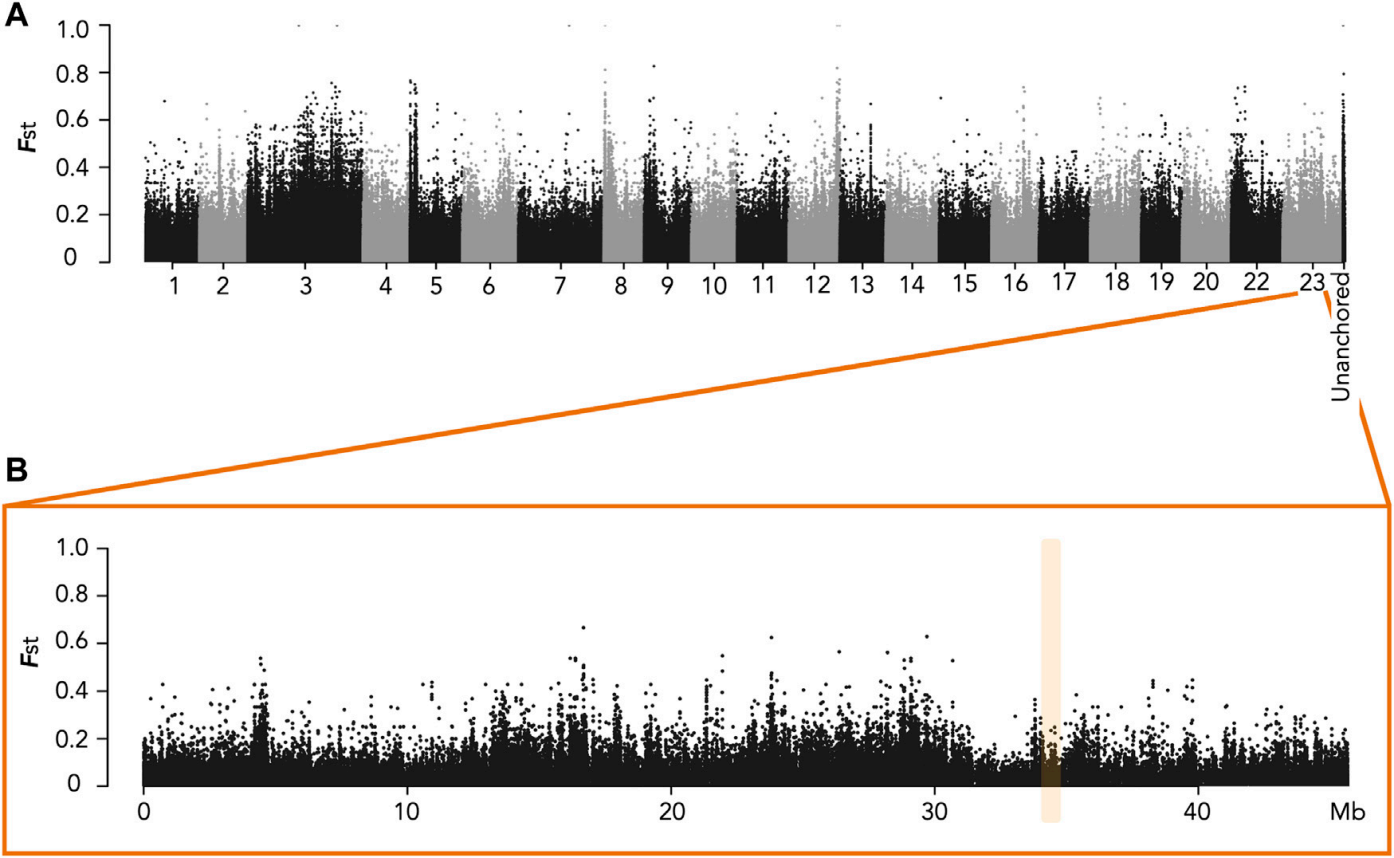
Manhattan plots of Fst for sites fixed or nearly fixed for one sex and at intermediate frequencies for the other sex in the Hora population. **(A)** Plot for all Linkage Groups. Unanchored contigs were grouped into a single label. **(B)** Plot of LG23 with the *amh* region highlighted in yellow. No high *F*st was evidenced in the expected *amh* region.

We had previously shown that males had a specific duplication of the *amh* gene (*amhY*/*amhΔY*) on LG23 in the Ethiopian Koka population as well as in the Ghanaian Kpandu population (Triay et al., 2020). We wanted to know if this was also the case in the Ethiopian Hora population. For this, we performed a short read WGS of wild-caught Hora males and females to search for strong sex-biased SNPs between sexes through a genome wide association study (GWAS). We obtained from this sequencing between 108,570,962 and 129,028,644 total reads, representing a mean of 17X per sample. We pooled by sex all the female reads and all the male reads, and then used for our analysis a random subset of 20% of the reads for each pool. The Manhattan plot of ***F*** st (Figure 2) did not reveal any obvious sex pattern on LG23. In the *oaz1* to *dot1l* region we found only one SNP in a non-coding region that differed between males and females. Furthermore, we did not see any reliable signal of sex-specific heterozygosity on other linkage groups for this population.

We also did not find evidence of a B chromosome. We did not observe any of the 16 shared regions of high coverage that potentially belong to a B chromosome, which we had previously found in Kpandu males and Koka females (Triay et al., 2020). Thus, the noisy ***F*** st signal is not from the presence of B chromosomes. Further male/female comparisons will be needed to determine the significance of the high ***F*** st peaks along the genome.

### 3.2 No Y Specific Reads in Hora Males in the Expected LG23 Sex-Linked Region

**FIGURE 3:**
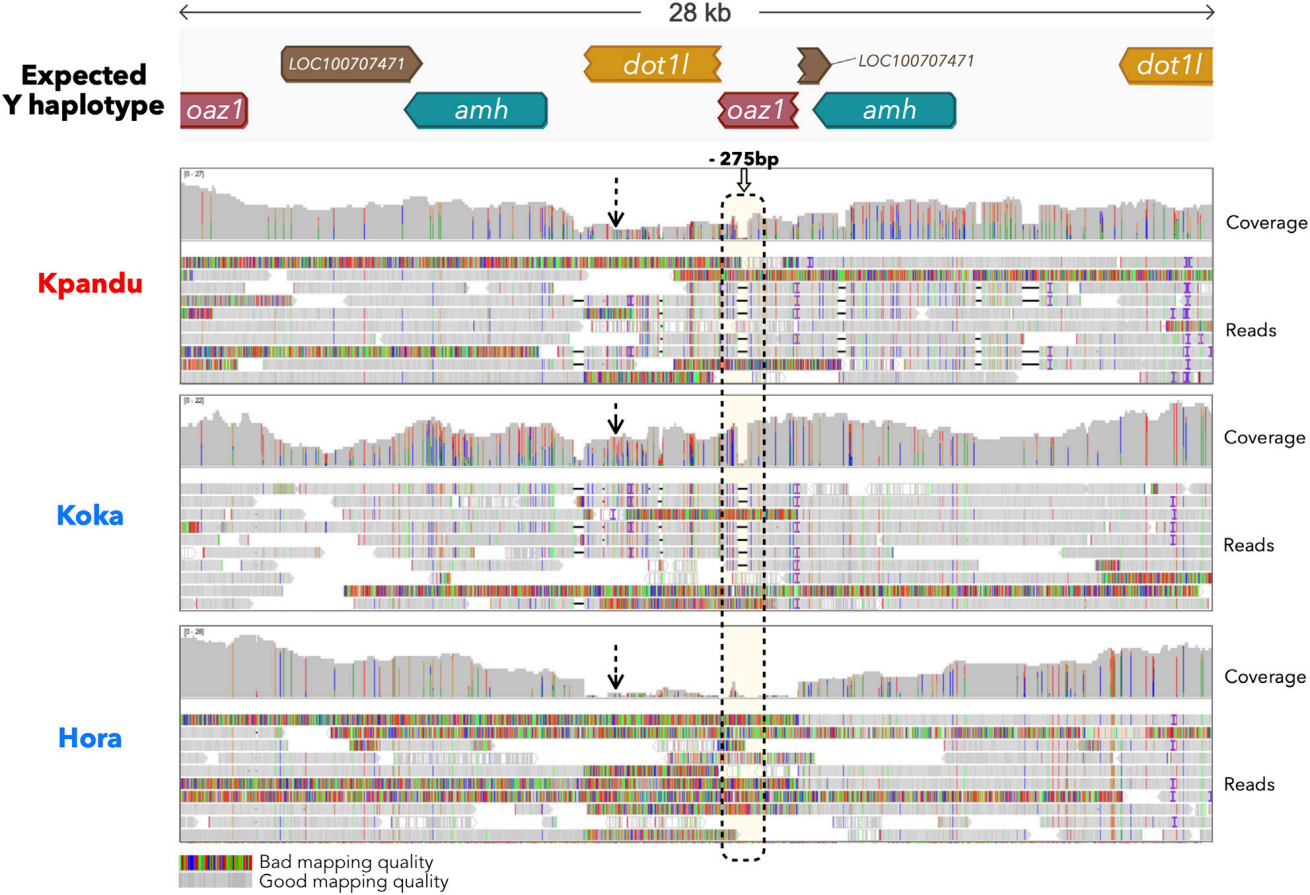
IGV snapshot of long reads mapping obtained from the Kpandu (Lake Volta, Ghana) male, the Lake Koka (Ethiopia) male and the Lake Hora (Ethiopia) male. Reads were mapped onto a Y expected haplotype. Dashed arrows highlight the coverage level over the beginning of the Y specific region. Reads mapping to the *oaz1-dot1l* chimera presenting a 275 bp deletion found in the *oaz1* truncated copy (within the dashed rectangle) were labelled as Y-specific.

We next performed a long-reads nanopore sequencing to study in Hora males in more depth the region around the *amh* gene that is duplicated and male-specific in the Koka and Kpandu populations. We sequenced on a MinION a male from each population. We obtained for the Hora male a total of 777,765 long nanopore reads corresponding to 4,034,783,321 bp, 4,474,365 reads corresponding to 22,830,560,109 bp for the Kpandu male and for the Koka male a total of 5,266,388 reads corresponding to 14,776,650,374 bp. The average error rate was 6.44%. This resulted in 228 reads of Kpandu, 287 of Koka and 236 reads of Hora that passed the *oaz1*, *amh* and *dot1l* BLAST filter. The expected Y haplotype structure we constructed is correct since Kpandu and Koka reads mapped to the Y haplotype with high quality, confirming the structure we previously described using WGS short reads data (Triay et al., 2020). However, the coverage was different along this region for the three males. It appeared to be almost constant along the region for the Kpandu and Koka males, but approached 0 in the Hora male (Figure 3). We found no reads in the Hora male that had a good mapping score over the Y specific regions. Moreover, the Hora male presents none of the expected Y specific indels (such as the 275 bp deletion in the truncated copy of the *oaz1* gene). This confirms that this Hora male does not carry any tandem duplication in this region and that the *amh* duplicated region is most likely not the sex-determining region in the Hora population.

### 3.3 Assembly of the Sex Haplotypes

For the Kpandu and Koka males we considered the reads containing a deletion of 275 bp in the truncated *oaz1* copy as “Y-specific” reads. The reads not containing this deletion were considered as belonging to the X chromosome. This resulted in 15 long reads that were considered Y-specific for the Kpandu male and 11 for the Koka male. In order to obtain the Y haplotype and study it in more detail, our first approach was to do a *de novo* assembly of the long reads from the sex-region. The contigs obtained were compared to the *O. niloticus* XX reference genome (a female from the Stirling-Manzala strain). The automatic annotation of contigs based on the reference genome and the expected Y haplotype allowed us to label the different contigs as X or Y haplotypes.

**FIGURE 4:**
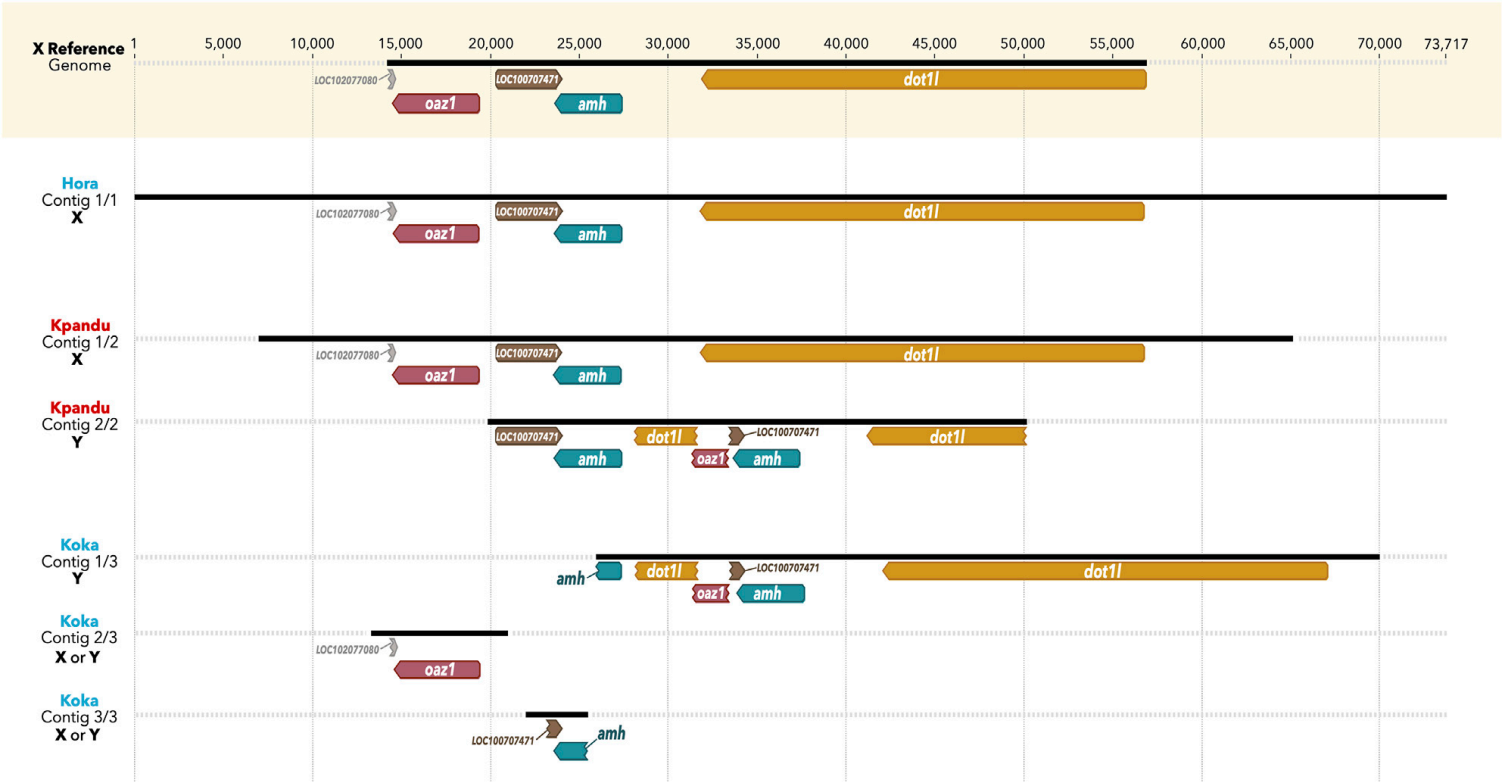
Contigs output of the Canu *de novo* assembly of the three males belonging to the wild populations of Hora, Koka and Kpandu. Koka and Hora (in blue) are Ethiopian males from lakes of the same names, whereas Kpandu (in red) is from Lake Volta in Ghana. All contigs are visually aligned to the *oaz1* gene of the reference genome, based on Geneious automated annotations from the XX reference genome and from the expected Y haplotype built previously. X and Y labels of contigs are deduced according to the structure of contigs, presenting or not the *dot1l-oaz1* chimera, specific to the Y haplotype as described in Triay et al. (2020).

We observed only one long contig for the Hora male, consistent with an X haplotype (Figure 4). No Y haplotype was assembled, in agreement with the fact that the mapping results revealed no reads presenting Y-specific structures. In contrast, two contigs were assembled for the Kpandu male: one perfectly corresponding to the X reference genome haplotype, and another one presenting the Y-specific structures. The second contig however is much smaller than the X haplotype and is centered around the *oaz1-dot1l* chimera and a second copy of the *amh* gene [corresponding to the *amhΔY* described in the Japanese strain (Li et al., 2015)]. Finally, three contigs were built from the Koka reads. The longest contig carries Y-specific structures, such as the *dot1l-oaz1* chimera and the Y-specific indels corresponding to the duplicated region. However, the first copy of the *amh* gene (which is the *amhY* in the Japanese strain) is not as fully reconstructed as for the Kpandu male. Two smaller contigs contain respectively the conserved version of the *oaz1* gene and the beginning of the *amhY* gene. No clear X haplotype was built from the *de novo* using all reads for the Koka male. *De novo* assemblies of “Non-Y specific” reads did not produce any contig long enough to be associated to the X haplotype either. Moreover, the longest contig assembled using the non Y-specific reads was identified as belonging to the Y haplotype as part of the chimera that could be identified with the automated annotation. Although a Y haplotype is present in the Koka assembly output, we did a *de novo* assembly with a pairwise alignment method to obtain a longer Koka Y contig.

The alignment of the Kpandu and Koka Y haplotypes obtained from MinION long reads together with the X haplotypes from the WGS short reads showed that the large structure corresponding to the tandem duplication of *oaz1-amh-dot1l* genes was well aligned between the two Y haplotypes, and that it was absent in all X haplotypes. This confirmed once again the existence of the Y structure as suggested. The low coverage of the MinION long reads however did not allow us to correct for the sequencing errors or ambiguities, and although it is very informative for the duplicated region it did not allow us to perform SNP or phylogenetic analyses with these Y haplotypes.

### 3.4 Comparing Sex Haplotypes to Population Phylogeny

**FIGURE 5:**
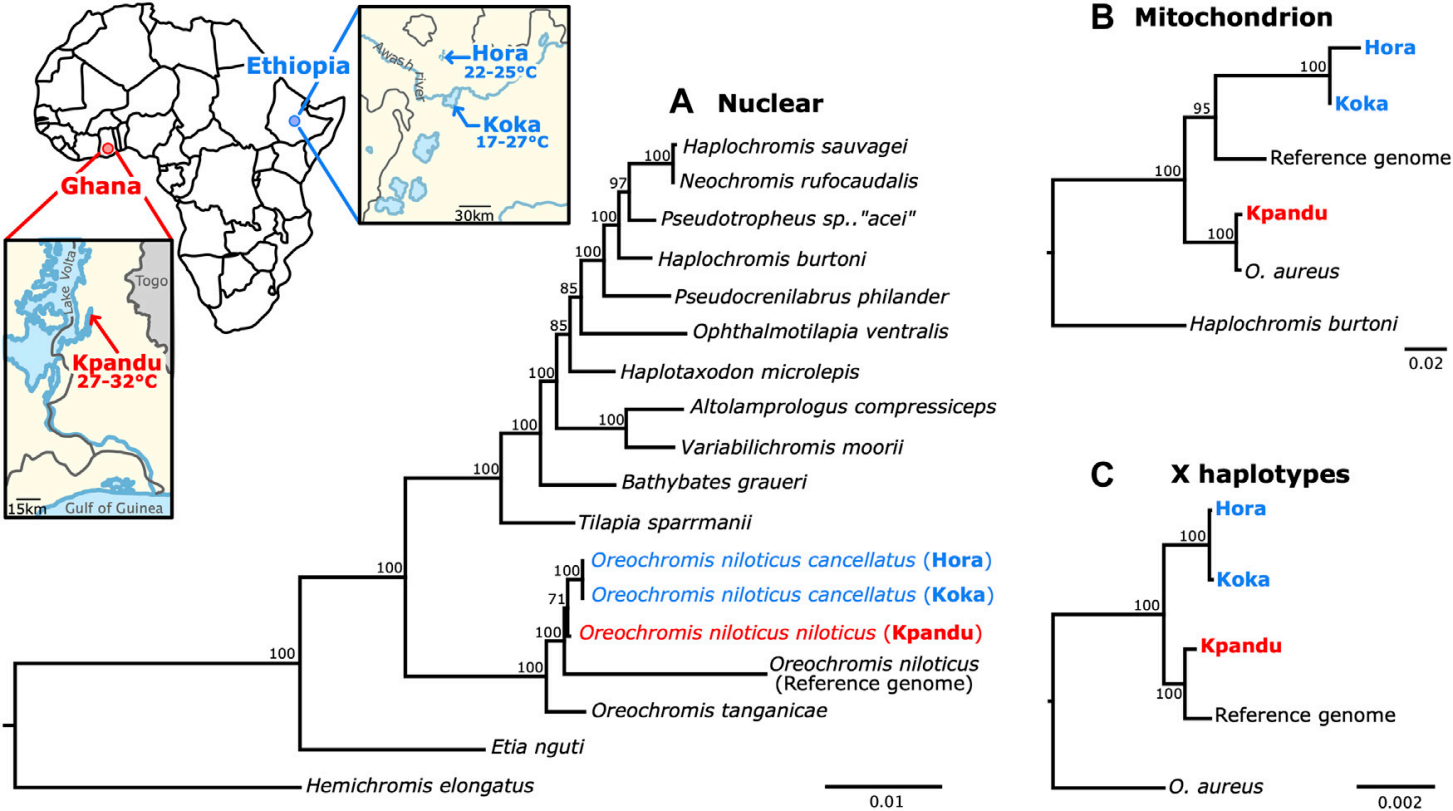
Phylogenetic analysis of Nile tilapia populations and related cichlids built with the maximum likelihood method. Each node of the tree corresponds to bootstrap values. Koka and Hora (in blue) are Ethiopian populations of *O. niloticus cancellatus* and Kpandu (in red) is a Lake Volta population of *O. niloticus niloticus* originating from Ghana. **(A)** Nuclear DNA phylogeny built using the 42 loci from Meyer et al. (2015). The *Oreochromis* species are forming a monophyletic clade in which the three wild populations are more closely related to each other than to the individual used from the reference genome (Manzala-Stirling strain). The tree is rooted with *Hemichromis elongatus*. The species from Meyer et al., 2015 are positioned identically to their phylogeny. These results are also showing that the *O. niloticus cancellatus* Koka and Hora populations are more closely related to each other than to the *O. niltocus niloticus* of Kpandu population. **(B)** Mitochondrial DNA phylogeny built from 13 mitochondrial protein-coding genes and the two ribosomal subunits. The tree is rooted with *Haplochromis burtoni*. This phylogeny is confirming that Koka and Hora populations are more closely related to each other than to Kpandu population. *O. niloticus* samples from Kpandu population are forming a monophyletic clade with *O. aureus*. **(C)** LG23 X haplotype phylogeny from LG23 *oaz1* to *dot1l* genes. The tree is rooted with *O. aureus.* Koka and Hora *O. niloticus* populations are forming a monophyletic clade

To establish the phylogenetic relationships among the three populations studied, we constructed phylogenetic trees based on nuclear genes, on the mitochondrial genome and finally on the X haplotype. Both the nuclear and the mitochondrial phylogenies showed that the two Koka and Hora populations from Ethiopia are more closely related to each other than to the Kpandu population from Ghana (Figure 5). These results are consistent with the geographical location of these three populations as well as with the subspecies taxonomy since both Koka and Hora are populations of *O. niloticus cancellatus* while the Kpandu population belongs to the subspecies *O. niloticus niloticus* (Trewavas, 1983; Bezault et al., 2011). The mitochondrial phylogeny also strongly supports a sister clade relationship between the Kpandu *O. niloticus* population and the *O. aureus* individual. The X haplotypes of Ethiopian populations also form a monophyletic group, with the Ghanaian Kpandu population as a sister group. In X haplotypes, the divergence between Hora and Koka females is hardly noticeable. Surprisingly, the position of the Kpandu population is different in the three trees. According to the mitochondrial data, Kpandu branches closely to *O. aureus*, as a sister group to all other *O. niloticus* sampled here. In the X haplotypes’ phylogeny, the Kpandu population is a sister clade to the reference genome, a tree topology that might be due to the Manzala-Stirling stock (from which was sampled the individual sequenced for the reference genome) originating from the Nile Basin. This basin was isolated from the Sudano-Sahelian region comprising the Kpandu population by several paleo-geographic events (Bezault et al., 2011). However, this result does not stand when studying a wider proportion of the genome, as the analysis of a concatenation of 42 nuclear exons yields a tree where Kpandu is a sister-clade to the Hora and Koka populations although the support for this relation is the weakest in the tree (with a bootstrap support of 71).

## 4 Discussion

Among populations of *O. niloticus* the sex determining locus has been found on LG1 **(** Lee et al., 2003; Cnaani et al., 2008; Gammerdinger et al., 2014; Palaiokostas et al., 2015) or on LG23 (Eshel et al., 2014; Li et al., 2015), or ob both (Taslima et al., 2021). The most prevalent and widespread sex chromosome is LG23 since it has been found now in numerous domesticated strains (Eshel et al., 2014; Li et al., 2015; Wessels et al., 2017; Cáceres et al., 2019; Curzon et al., 2020; Taslima et al., 2020) and also in wild populations (Sissao et al., 2019; Triay et al., 2020). We have previously shown that the *amhY* and *amhΔY* tandem duplication on the Y haplotype of LG23 is found in males of the two wild populations of Kpandu (from the Volta Lake in Ghana) and Lake Koka (in Ethiopia) (Triay et al., 2020). Because of the distant relationship of Kpandu-Volta in West Africa and Koka populations in East Africa (Bezault et al., 2011), we presume that the male-specific region is most likely ancestral in the *O. niloticus* species. Hence, we expected to find the same male-specific region on LG23 in another closely related population living in Lake Hora also from Ethiopia. Surprisingly, our short read genomic sequencing of Hora males and females revealed no association of sex with LG23 (Figure 2). Furthermore, among a total of ten wild males studied, no evidence of a duplication was observed in the alignment of the *amh* region. These results indicate that the LG23 Y is either segregating in very low frequencies and is no longer the major sex determinant in the Hora population or that it could have been lost entirely.

In our previous study, we predicted a Y haplotype based on the analyses of short reads of the duplicated region in males of the Koka and Kpandu populations (Triay et al., 2020). Assemblies of the Nanopore long reads, aided by this expected Y haplotype, allowed us to cross the duplicated region encompassing the two *amh* genes on LG23. We were able to confirm the presence of this Y-specific duplication, its structure and the order of genes inside this region, in wild populations from Lake Koka in Ethiopia and Lake Volta in Ghana (Figure 3). In both populations, the duplication occurs within the *oaz1* and *dot1l* genes, and contains truncated copies of *dot1l* and *oaz1* upstream of the *amhY* promoter. Kpandu males from Lake Volta have a similar structure and the same indels as the males from the Japanese strain analyzed by Li et al. (2015). One of the *amh* genes on the Y of Kpandu males resembles the truncated *amhΔY* copy found in the males from the Japanese strain (Li et al., 2015) since it also has the ∼233 bp deletion in the last exon 7 as well as the 5 bp insertion of exon 6 that causes a premature stop codon. Therefore, the other Kpandu *amh* gene is presumably the SD *amhY* gene but it does not have the missense SNP found in the Japanese strain (Li et al., 2015). In contrast, the two *amh genes* in Koka males do not have neither the missense SNP of *amhY*, nor the indels within *amhΔY*, particularly the 5 bp insertion responsible for a truncated gene. Consequently, both the *amhY* and *amhΔY* of Koka males might be functional and may play a role in maleness. In Hora males however, no Y haplotype could be constructed from the long reads (Figures 3, 4). The assemblies of Hora males revealed that they had a single copy of *amh* similar to the X haplotype, and confirmed that there is no male-specific duplication around the *amh* region on the LG23. Hora males could therefore have lost the sex determining region and LG23 is likely no longer the sex chromosome.

Regarding the rest of the genome, although we found an overall high *F*st throughout the genome, we found no significant association of sex with other LGs. In particular, we did not find an association with LG1in Hora individuals. Sex segregated for LG1 in some domesticated strains from US commercial stocks (Gammerdinger et al., 2014), in a Ghanaian family (Cnaani et al., 2008) and in the Manzala-Stirling strain (Palaiokostas et al., 2015). In this last strain, LG23-Y is still segregating at low frequencies (Taslima et al., 2021), although sex maps to LG23 in both the Manzala-Göttingen stock (Wessels et al., 2017) and the Manzala-Tihange stock (Sissao et al., 2019) which originated from the Manzala-Stirling stock. No wild population studied to date shows sex segregation to LG1. It is plausible that LG1 might have become the pair of sex chromosomes under conditions such as domestication inducing gene loss or fixation by drift. The fact that this individual presents such a long branch in our phylogenies reinforces the idea of a strong molecular divergence of this stock. This might have occurred during gynogenesis to produce the homogygous female clonal line from which the reference genome individual belonged (Sarder et al., 1999). Change in SD system with the loss of the sex chromosome occurred in laboratory stocks of zebrafish (Wilson et al., 2014). Alternatively, this molecular divergence of the Manzala-Stirling strain could result from a hybridization that might have happened during the process of domestication, which could explain the large divergence observed with other *O. niloticus* populations. This could even be the cause of the emergence of a new sex-determining system on LG1. For instance, the Thai- Chitralada hybrid, a strain originating from crosses of *O. niloticus, O. mossambicus* and *O. aureus*, presents sex-associated markers on LG1, LG3 and LG23 (Curzon et al., 2019). An introgression of closely related species of *O. niloticus* could thus favor the turnover of sex-chromosomes so that LG1 became fixed as a new sex chromosome.

The absence of the Y *amh* duplication in the Hora Lake population reflects a rapid change in SD within Nile tilapia populations. This is another example of the rapid turnover of SD within the cichlid family. Indeed, more than 20 different major sex-determining genes, on more than 17 LGs, have now been described in cichlids, highlighting the fact that in this family there are a great diversity of sex determining systems that have emerged between closely related species (Gammerdinger and Kocher, 2018; El Taher et al., 2021; Tao et al., 2021). To date, cichlids from Lake Tanganyika, appear to have the highest rates of sex chromosome turnover as well as transitions between male and female heterogamety (El Taher et al., 2021).

Our study emphasizes the need to analyse the evolution of sex chromosomes at the population level. Studies in Nile tilapia have demonstrated that in domesticated strains (Wessels et al., 2014; Taslima et al., 2021) and wild populations (Bezault et al., 2007; Sissao et al., 2019) the sex-determination system can be more complex than just the *amh* gene on LG23. Sex in the Nile tilapia is genetically inherited through the sex chromosomes but there is also a genetic inheritance of autosomal factors and of temperature sensitivity (Baroiller and D’Cotta, 2001; Bezault et al., 2007; Baroiller et al., 2009; Wessels et al., 2017). Temperature could facilitate the transition to a new sex chromosome/determination system (Grossen et al., 2011; Baroiller and D’Cotta, 2016). This, along with the results of the Hora population lacking the LG23 Y sex-determinant suggest that the turnover in sex chromosomes and sex-determination systems might not be rare events at the intraspecific level. Variance in sexual conflict and recombination patterns, two important factors possibly for the evolution of sex chromosomes, could induce divergence between sex chromosomes of different populations.

The absence of the Y chromosome on LG23 in Hora raises the question of why the Hora population has experienced a turnover in the system of sex determination. Causes for sex chromosome turnovers can be numerous, including a loss of the sex-determinant by drift, sex ratio bias (e.g. environmental effects inducing sex-reversal), or pleiotropic effects of a sexually antagonistic mutation (Tao et al., 2021). Demographic events might have caused changes in Lake Hora SD. Lake Hora is thought to have been restocked at the beginning of the 20th century with the endemic subspecies *O. n. cancellatus* (Bezault, 2005). Lake Hora is, nevertheless, a small lake (∼1.15 km^2^) and the effective population size is supposedly small. The Hora population might have experienced a genetic bottleneck that led to the loss of the major sex-determinant region on LG23-Y by drift. Intraspecific differences in SD was also observed for the haplochromine cichlid *Pseudocrenilabrus philander* for which WGS detected a strong XY locus in LG7 in a lake population whereas no GSD was found in the outflow creek population which experienced genetic bottleneck (Böhne et al., 2019).

Sex ratio bias can also occur in *O. niloticus* species because of the effects of environmental temperature. This species can undergo a sex reversal over the threshold temperature of 32°C, causing XX individuals to develop as viable and fertile males. This temperature sensitivity is a variable and heritable trait (Bezault et al., 2007; Wessels and Hörstgen-Schwark, 2007; Baroiller et al., 2009; Wessels and Hörstgen-Schwark, 2011). In the most extreme scenario, the increasing number of XX individuals (males and females) in a population could progressively lead to the loss of the Y chromosome and allow a new master gene to control sex-determination in the population. XX males have been found in many wild populations living in masculinizing temperatures above 32°C such as Lake Metahara in Ethiopia (Bezault et al., 2007; Baroiller et al., 2009). Lake Hora is referred to as a “cold” lake because its temperature varies between 17 and 26°C during the year, which is well below the masculinizing temperature. Nevertheless, Lake Hora might have been restocked with a high proportion of XX males. Hence, the loss of an ancestral LG23 Y system in the Hora fish could have been facilitated by demographic events of this small population, with colonization of XX neo-males and perhaps influenced by founder effects of a small number of females.

What could the current sex determining system(s) of the Hora population be? When a major sex-determinant is lost, it can be replaced by a new “master” gene, possibly already implicated in the sex-determination and differentiation pathway. In the case of the Hora population, we did not identify any obvious sex-determinant loci when using whole genome sequencing of wild male and female pools. This could be due to the new major sex-determinant region being too recent to have accumulated enough SNPs to be detected through genome wide *F*st analysis. It is also possible that after the loss of the major determinant LG23 Y, other loci involved in sex determination and differentiation pathway acquire a more important role and coexist during a transition period until the fixation and potential appearance of a new major determinant (Furman et al., 2020; Tao et al., 2021). In this case, more than one locus is segregating on several chromosomes, appearing as a polygenic sex determining system (Mank and Avise, 2009; Roberts et al., 2009). Although WGS of pooled sexes is a very efficient method to quickly detect sex determination locus and the linkage group acting as a sex chromosome when the system is monogenic, it is not as efficient to detect polygenic systems (Gammerdinger and Kocher, 2018). We cannot exclude the scenario of two sex-determining loci (or more) segregating in the Hora population as recently suggested by (Taslima et al., 2021) for the Manzala-Stirling stock, and evidenced in several cichlids (Tao et al., 2021). Another alternative is that the SD in the Hora population has suffered a transition from a genetic to an environmental sex determination system as recently demonstrated in a ZZ/ZW lizard (Holleley et al., 2015). Association of other genomic approaches, other than sex-pooled WGS, with more alignments strategies will be necessary to search further for the sex determination locus or sex chromosome(s) in Hora fish.

The X and Y chromosomes of the Koka and Kpandu wild populations of *O. niloticus* studied here are visually homomorphic, like all cichlids sex chromosomes karyotyped to date (Gammerdinger and Kocher, 2018). Reduced sex chromosome differentiation is evidenced by the small size of the sex-specific region (<0.5 Mb) of LG23 suggesting that recombination still occurs around the sex-region. The study of genotype-phenotype associations using ddRAD methods on families of Kpandu and Koka populations already suggested that recombination could occur close to the male-specific region (Triay et al., 2020). This reduced recombination further highlights the fact that LG23 has remained a “young” pair of sex chromosomes. The shared structure between males of the two wild populations suggests this XX/XY system predates the divergence of populations of *O. niloticus.* Thus, even if chronologically old, Nile Tilapia sex chromosomes do not seem to have evolved to a non-recombinant Y that starts to differentiate, but is maintained at a state of low recombination for long evolutionary times.

Our phylogenetic studies confirmed that the Ethiopian Koka and Hora populations, both from the subspecies *Oreochromis niloticus cancellatus,* belong to a monophyletic clade with the Kpandu population *O. niloticus niloticus* branching as a sister group. These results appear contradictory with a shared ancestral Y chromosome between Koka and Kpandu males that is not shared between Koka and Hora. Microsatellite analysis showed that the Koka population of the Ethiopian Awash basin is genetically different at the macrogeographical level from the Kpandu population located in the Sudano-Sahelian region (Bezault et al., 2011). The phylogeny we inferred is in accordance with this previous study: the subspecies *O. n. cancellatus* found in Lake Koka and Lake Hora in the Ethiopian basin are sister populations, reflecting the genetic proximity seen at the micro-geographic level (Bezault et al., 2011), while the Kpandu population is more divergent. It is parsimonious to think that the Y *amh* duplication on LG23 is ancestral since it is found in Nile tilapia populations from two distant African hydrographic basins. Nevertheless, to better understand the evolutionary history of the LG23 Y chromosome, phylogenies including additional populations are required. It would be important to add populations from the Nile region because fish from Lake Manzala (Egypt) might carry a sex locus on LG1. It would also be important to add other populations from the Ethiopian Rift Valley. Inferring a phylogeny with several of these populations and checking for the presence/absence of the *amh* duplication on LG23 Y would enable us to date the event that led to the apparent loss of the LG23 Y in the Hora population.

We noted an incongruence for the Kpandu position between the nuclear, mitochondrial and the X haplotypes’ phylogenies (Figure 5). Our mitochondrial DNA analysis demonstrates a clustering of Kpandu *O. niloticus* population with the sample of *O. aureus,* also a Nilo-Sudanic species. The formation of a monophyletic group is consistent with the study of Rognon and Guyomard, (2003) in which they hypothesize a complete introgression of the mitochondrial DNA from *O. aureus* to *O. niloticus* in Western African populations, but also in Egyptian lakes. Clustering of Kpandu Nile tilapia and *O. aureus* was also observed when using mtDNA but they were differentiated in phylogenetic trees derived from nuclear DNA markers (SNPs) (Syaifudin et al., 2019). Discordance between mtDNA and nuclear DNA has been shown in several studies within the *Oreochromine* cichlids (Ford et al., 2019). Mitochondria DNA reflects only maternal inheritance, whereas species topology is probably more reliable when using multi-nuclear markers (Meyer et al., 2015; Ford et al., 2019). Ancient hybridization was suggested as being the most likely explanation for the discordance between mtDNA and nuDNA (Dunz and Schliewen, 2013). If this is the case, our mitochondrial analysis is not suitable to infer the relative position of Kpandu to our other *O. niloticus* samples. By contrast, our nuclear DNA datasets should not be affected by mitochondrial introgressions. However, we observed two possible branchings of O. *niloticus* samples. In our phylogeny built with the X haplotypes, Kpandu groups with the reference genome while this population is a sister-clade to the Ethiopian populations Hora and Koka in the nuclear exon tree. These conflicting signals may result from a methodological bias. Indeed, the nuclear exons’ phylogeny was inferred using more data than the X haplotype analysis, sampled from 42 different loci in the genomes. This methodology should alleviate sampling errors due to loci-specific events, such as incomplete lineage sorting or GC-biased gene conversion (Degnan and Rosenberg, 2009), even if it does not completely resolve related difficulties (Philippe et al., 2011). On the other hand, we see that this node is the least supported in the tree, hinting that this incongruence may be due to a phenomenon affecting a large proportion of the genome. Thus, a biological explanation such as hybridization with other strains may also explain this discrepancy. Moreover, the individual of *O. niloticus* used for the reference genome (a XX female belonging to a gynogenetic homozygous clonal line generated at the University of Stirling (Sarder et al., 1999) shows a longer branch than all other samples in the three trees, which may also be the result of admixture with another more distantly related population. Proper admixture analyses could help untangle this relationship between the Kpandu population and the reference genome.

Our phylogenetic results suggest that at some point in the evolutionary history of *O. niloticus* introgression with other closely related cichlids species might have occurred. *O. niloticus* might have hybridized in the past or recently due to aquaculture practices with other closely related species since several species coexist in many African fresh water basins. *O. aureus* and *O. niloticus* divergence was estimated to be 2.93 MYA (Xiao et al., 2015). They cohabitate in several river basins, both being Nilo-Sudano species (Trewavas, 1983), but they are not known to hybridize naturally (Payne and Collinson, 1983). In Lake Manzala there is a question on whether hybridization might have occurred between Nile tilapia and *S. galilaeus* confused perhaps as *O. aureus* (Ford et al., 2019). This might be the explanation of a SD on LG1 appearing in several *O. niloticus* strains (Cnaani et al., 2008). Thus, particularly when studying sex chromosomes, it might be essential to consider the other sex-determining loci segregating in the sympatric species of studied populations.

## 5 Conclusion

In this work, we sequenced the whole genome of a Nile tilapia from Lake Hora, Ethiopia. The short read analysis revealed the absence of sex-linked sequences on LG23 and we were unable to find a strong association of sex with any region of the genome in this population. Using long reads, we reconstructed a Y haplotype corresponding to a duplicated male-specific region on the LG23 sex chromosome in populations from Lake Volta (Ghana) and Lake Koka (Ethiopia). This male-duplication of LG23 spans ∼51 kb and is likely to be ancestral to the divergence of Eastern and Western African populations. In contrast, the *amh* male-specific duplication could not be detected in the Hora males using either WGS or nanopore long reads, suggesting that this population has lost the LG23 Y chromosome. This work highlights that turnover of the sex-determination system can occur rapidly even between closely related populations in the *O. niloticus* species. One hypothesis is that sex determination in the Hora population might be an allelic diversification on another LG, or alternatively sex determiners might be segregating on several LGs in a polygenic SD system. Another alternative is that the Hora population have acquired an ESD system. Further analysis will be required to understand the sex-determination system in the Hora population and potentially find the major sex-determination gene.

## Data Availability

The datasets presented in this study can be found in online repositories. The names of the repository/repositories and accession number(s) can be found below: National Center for Biotechnology Information (NCBI) BioProject database under accession number PRJNA802819. The raw Hora WGS Illumina sequencing data are under accession numbers SAMN25598580 to SAMN25598599. The data of the raw MinION nanopore long reads are under accession numbers SAMN25610565 to SAMN25610567.
