# Supplementary material for: Polymorphism of Sex Determination Amongst Wild Populations Suggests its Rapid Turnover Within the Nile Tilapia Species
Source: Front Genet. 2022 May 17;13:820772. doi: 10.3389/fgene.2022.820772 (PMC9152217; doi:10.3389/fgene.2022.820772)
Supplement: Supplementary file 1 [file DataSheet2.PDF]

Supplementary Table 2. Mitochondrial sequences used to infer the phylogeny. The 13 mitochondrial protein-coding genes and large and small ribosomal subunits (rrnL and rrns respectively) alignments length are shown.

Gene	Alignement length (bp)
ATP6	184
ATP8	168
COX1	1596
COX2	699
COX3	783
CYTB	1140
ND1	975
ND2	1047
ND3	351
ND4	1386
ND4L	297
ND5	1959
ND6	642
rrnL	1821
rrnS	1005
