# Supplementary material for: Polymorphism of Sex Determination Amongst Wild Populations Suggests its Rapid Turnover Within the Nile Tilapia Species
Source: Front Genet. 2022 May 17;13:820772. doi: 10.3389/fgene.2022.820772 (PMC9152217; doi:10.3389/fgene.2022.820772)
Supplement: Supplementary file 2 [file DataSheet1.PDF]

Supplementary Table 1. Genes used to infer the nuclear phylogeny. Gene nomenclature, species and accession numbers are excerpted from Meyer et al., 2015.

Species / Gene ID	Altolamprologus compressiceps	Bathybates graueri	Haplochromis burtoni	Haplotaxodon microlepis	Neochromis rufocaudalis	Ophthalmotilapia ventralis	Oreochromis tanganicae
**rag1**	KP131329	KP131317	KM263618	KP131326	KP131331	KM263621	KP131316
**b2m**	KP129986	KP129970	KP129955	KP129983	KP129989	KP129959	KP129969
**gapdhs**	KP130246	KP130234	KM263633	KP130243	KP130248	KM263636	KP130233
**Ptchd4**	KP131371	KP131354	KP131338	KP131368	KP131374	KP131342	KP131353
**enc1**	KP131215	KP131198	KP131182	KP131212	KP131218	KP131186	KP131197
**phpt1**	KP130420	KP130403	KP130388	KP130417	KP130422	KP130392	KP130402
**rps7**	KP129946	KP129934	KM263648	KP129943	KP129948	KM263651	KP129933
**tbr1**	KP130121	KP130104	KP130088	KP130118	KP130124	KP130092	KP130103
**aqp1a.1**	KP131036	KP131020	KP131004	KP131033	KP131039	KP131008	KP131019
**hprt1**	KP130992	KP130976	KP130960	KP130989	KP130995	KP130964	KP130975
**anxa4**	KP130031	KP130014	KP129998	KP130028	KP130034	KP130002	KP130013
**pgk1**	KP129879	KP129862	KP129846	KP129876	KP129882	KP129850	KP129861
**bmp4**	KP130912	KP130900	KM263663	KP130909	KP130914	KM263666	KP130899
**bmp2**	KP129756	KP129739	KP129723	KP129753	KP129759	KP129727	KP129738
**TMO-4C4**	KP130705	KP130688	KP130672	KP130702	KP130708	KP130676	KP130687
**fgf6b**	KP130076	KP130059	KP130043	KP130073	KP130079	KP130047	KP130058
**runx2**	KP131416	KP131399	KP131383	KP131413	KP131419	KP131387	KP131398
**furina**	KP130750	KP130733	KP130717	KP130747	KP130753	KP130721	KP130732
**wnt7b**	KP130166	KP130149	KP130133	KP130163	KP130169	KP130137	KP130148
**pax9**	KP130333	KP130316	KP130300	KP130330	KP130335	KP130304	KP130315
**sox10b**	KP129711	KP129695	KP129679	KP129708	KP129714	KP129683	KP129694
**otx2**	KP130464	KP130447	KP130431	KP130461	KP130467	KP130435	KP130446
**otx1**	KP131081	KP131064	KP131048	KP131078	KP131084	KP131052	KP131063
**dlx2a**	KP130551	KP130535	KP130519	KP130548	KP130554	KP130523	KP130534
**dlx4b**	KP131293	KP131276	KP131260	KP131290	KP131296	KP131264	KP131275
**barx1**	KP130628	KP130612	KP130596	KP130626	KP130631	KP130600	KP130611
**ednrb1a**	KP131251	KP131239	KM263678	KP131248	KP131253	KM263681	KP131238
**mc1r**	KP130288	KP130271	KP130255	KP130285	KP130291	KP130259	KP130270
**skia**	KP130376	KP130359	KP130343	KP130373	KP130379	KP130347	KP130358
**kita**	KP129801	KP129784	KP129768	KP129798	KP129804	KP129772	KP129783
**mitfa**	KP129837	KP129825	KM263693	KP129834	KP129839	KM263696	KP129824
**tyr**	KP129914	-	KM263708	KP129911	KP129916	KM263711	KP129902
**hagoromo (fbxw4)**	KP130587	KP130575	KM263723	KP130584	KP130589	KM263726	KP130574
**slc45a2 (aim)**	KP131126	KP131109	KP131093	KP131123	KP131129	KP131097	KP131108
**rh1**	KP130833	KP130816	KP130800	KP130830	KP130836	KP130804	KP130815
**opn1mw (lws)**	KP130509	KP130492	KP130476	KP130506	KP130511	KP130480	KP130491
**opn1sw (sws)**	KP130877	-	KP130845	KP130874	KP130880	KP130849	KP130860
**ccng1**	KP130950	KP130934	KP130920	KP130947	KP130953	KP130923	-
**snx33**	-	KP130776	KP130762	KP130788	KP130793	KP130766	KP130775
**rpl13a**	KP130210	KP130193	KP130178	KP130207	KP130213	KP130182	-
**edar**	KP131170	KP131154	KP131138	KP131167	KP131173	KP131142	KP131153
**csf1ra**	KP130663	KP130651	KM263738	KP130660	KP130665	KM263741	KP130650

Gene ID	Pseudocrenilabrus philander	Pseudotropheus sp	Tilapia sparrmanii	Tylochromis polylepis	Variabilichromis moorii
**rag1**	KM263622	KP131335	KP131337	KP131325	KP131314
**b2m**	KP129973	KP129994	KP129997	-	KP129968
**gapdhs**	KM263637	KP130252	KP130254	KP130242	KP130231
**Ptchd4**	KP131357	KP131379	KP131382	KP131366	KP131351
**enc1**	KP131201	KP131223	KP131226	KP131210	KP131195
**phpt1**	KP130406	KP130427	KP130430	KP130415	-
**rps7**	KM263652	KP129952	KP129954	KP129942	KP129931
**tbr1**	KP130107	KP130129	KP130132	KP130116	KP130101
**aqp1a.1**	KP131023	KP131044	KP131047	-	KP131017
**hprt1**	KP130979	KP131000	KP131003	-	KP130973
**anxa4**	KP130017	KP130039	KP130042	KP130026	KP130011
**pgk1**	KP129865	KP129887	KP129890	KP129874	KP129859
**bmp4**	KM263674	KP130918	-	KP130908	KP130897
**bmp2**	KP129742	KP129764	KP129767	KP129751	KP129736
**TMO-4C4**	KP130691	KP130713	KP130716	KP130700	KP130685
**fgf6b**	KP130062	KP130084	KP130087	KP130071	KP130056
**runx2**	KP131402	KP131424	KP131427	KP131411	KP131396
**furina**	KP130736	KP130758	KP130761	KP130745	KP130730
**wnt7b**	KP130152	KP130174	KP130177	KP130161	KP130146
**pax9**	KP130319	KP130340	KP130342	KP130328	KP130313
**sox10b**	KP129698	KP129719	KP129722	KP129706	KP129692
**otx2**	KP130450	KP130472	KP130475	KP130459	KP130444
**otx1**	KP131067	KP131089	KP131092	KP131076	KP131061
**dlx2a**	KP130538	KP130559	KP130562	-	KP130532
**dlx4b**	KP131279	KP131301	KP131304	KP131288	KP131273
**barx1**	KP130615	KP130636	KP130638	KP130624	KP130609
**ednrb1a**	KM263682	KP131257	KP131259	KP131247	KP131236
**mc1r**	KP130274	KP130296	KP130299	KP130283	KP130268
**skia**	KP130362	KP130384	KP130387	KP130371	KP130356
**kita**	KP129787	KP129809	KP129812	KP129796	KP129781
**mitfa**	KM263697	KP129843	KP129845	KP129833	KP129822
**tyr**	KM263712	KP129919	KP129921	KP129910	KP129900
**hagoromo (fbxw4)**	KM263727	KP130593	KP130595	KP130583	KP130572
**slc45a2 (aim)**	KP131112	KP131134	KP131137	KP131121	KP131106
**rh1**	KP130819	KP130841	KP130844	KP130828	KP130813
**opn1mw (lws)**	KP130495	KP130516	KP130518	KP130504	KP130489
**opn1sw (sws)**	KP130863	KP130885	KP130887	KP130872	KP130858
**ccng1**	KP130937	KP130958	-	-	KP130932
**snx33**	KP130779	KP130798	-	-	KP130774
**rpl13a**	KP130196	KP130218	KP130221	KP130205	KP130191
**edar**	KP131157	KP131178	KP131181	KP131165	KP131151
**csf1ra**	KM263742	KP130669	KP130671	KP130659	KP130648
